# Association of Existence of Sarcopenia and Poor Recovery of Swallowing Function in Post-Stroke Patients with Severe Deglutition Disorder: A Multicenter Cohort Study

**DOI:** 10.3390/nu14194115

**Published:** 2022-10-03

**Authors:** Shinta Nishioka, Ichiro Fujishima, Masako Kishima, Tomohisa Ohno, Akio Shimizu, Takashi Shigematsu, Masataka Itoda, Hidetaka Wakabayashi, Kenjiro Kunieda, Fumiko Oshima, Sumito Ogawa, Kazuki Fukuma, Nami Ogawa, Jun Kayashita, Minoru Yamada, Takashi Mori, Shinya Onizuka

**Affiliations:** 1 Department of Clinical Nutrition and Food Service, Nagasaki Rehabilitation Hospital, Nagasaki 850-0854, Japan; 2 Department of Rehabilitation Medicine, Hamamatsu City Rehabilitation Hospital, Hamamatsu 433-8511, Japan; 3 Department of Dentistry, Wakakusa-Tatsuma Rehabilitation Hospital, Daito 574-0012, Japan; 4 Department of Dentistry, Hamamatsu City Rehabilitation Hospital, Hamamatsu 433-8511, Japan; 5 Department of Health Science, Faculty of Health and Human Development, University of Nagano, Miwa 380-8525, Japan; 6 Department of Oral Rehabilitation, Osaka Dental University Hospital, Hirakata 573-1144, Japan; 7 Department of Rehabilitation Medicine, Tokyo Women’s Medical University Hospital, Shinjuku-ku, Tokyo 162-0054, Japan; 8 Department of Neurology, Gifu University Graduate School of Medicine, Gifu 501-1194, Japan; 9 Department of Rehabilitation Medicine, Japanese Red Cross Society Suwa Hospital, Suwa 392-8510, Japan; 10 Department of Geriatric Medicine, Graduate School of Medicine, The University of Tokyo, Bunkyo-ku, Tokyo 113-8655, Japan; 11 Department of Neurology, National Cerebral and Cardiovascular Center, Suita 564-8565, Japan; 12 Department of Dentistry, Sakaue Dental Office, Setagaya-ku, Tokyo 158-0094, Japan; 13 Department of Health Sciences, Prefectural University of Hiroshima, Hiroshima 734-8558, Japan; 14 Faculty of Human Sciences, University of Tsukuba, Bunkyo-ku, Tokyo 112-0012, Japan; 15 Department of Oral and Maxillofacial Surgery, Southern Tohoku General Hospital, Koriyama 963-8563, Japan; 16 Department of Rehabilitation Medicine, Nagasaki Rehabilitation Hospital, Nagasaki 850-0854, Japan

**Keywords:** activities of daily living, deglutition disorders, older adults, stroke rehabilitation, muscle mass, muscle strength

## Abstract

Background: The effect of sarcopenia on the recovery of swallowing function, and the interaction among sarcopenia, nutrition care, and rehabilitation therapy are inconclusive. Methods: This multicenter cohort study was conducted between November 2018 and October 2020 in convalescent rehabilitation hospitals in Japan and included post-stroke patients aged ≥65 years with dysphagia. All participants were assigned to sarcopenia and non-sarcopenia groups. The primary outcome was the achievement of ≥2 Food Intake Level Scale [FILS] gain, and the secondary outcomes included Functional Independence Measure (FIM) gain and efficiency. Considering the effect modification of energy intake and rehabilitation duration, logistic regression analyses were performed. Results: Overall, 153 participants with (median age, 82 years; 57.5% women) and 40 without (median age 75 years; 35.0% women) sarcopenia were included. The non-sarcopenia group had more patients who achieved an FILS gain of ≥2 (75.0%) than the sarcopenia group (51.0%). Sarcopenia was independently associated with a poor FILS gain (odds ratio:0.34, 95% confidence intervals: 0.13–0.86) but not associated with FIM gain or efficiency. This association was not affected by the rehabilitation duration or energy intake. Conclusions: In conclusion, sarcopenia was negatively associated with the recovery of swallowing function in stroke patients without interaction by energy intake and rehabilitation duration.

## 1. Introduction

Deglutition disorder is a common and life-threatening sequela following stroke. In one study, 23% of patients with ischemic stroke developed dysphagia [1], whereas a meta-analysis reported that the prevalence of oropharyngeal dysphagia varied widely, from 8.1% to 80%, depending on the diagnostic method [2]. Deglutition disorders can cause various complications, including aspiration pneumonia, dehydration, and nutritional deficit, and they are associated with increased short- and long-term mortality [3,4]. Several approaches, including behavioral, physical, and instrumental treatment, have been investigated to help patients regain swallowing function. Although some treatments affect swallowing function and result in a shorter length of hospital stay, no promising interventions to reduce mortality are available to date [5]. Effective strategies to treat dysphagia are yet to be established.

Recently, another potential etiology of dysphagia has gained increasing attention—sarcopenia in generalized and swallowing muscles [6,7,8]. The term “sarcopenic dysphagia” was first coined in 2012 [9]; thereafter, many studies examined the relationship between sarcopenia and dysphagia [10,11,12,13,14]. Current consensus on the diagnostic criteria of sarcopenic dysphagia encompasses the assessment of whole-body muscle mass and muscle strength and function, evaluation of swallowing function and tongue pressure, and eliminating other etiologies, such as stroke [15]. Dysphagia after stroke is distinguished from sarcopenic dysphagia in these terms.

However, several studies have determined an association between sarcopenia or reduced muscle mass and worsening swallowing function after stroke [16,17]. Sarcopenia after stroke (stroke-related sarcopenia) may impede the recovery of swallowing function [18], whereas pre-stroke sarcopenia may contribute to the prognosis of post-stroke dysphagia. Approximately 50% of stroke rehabilitation patients in Japan exhibit sarcopenia [19,20]; thus, the potential harmful effect of sarcopenia on swallowing rehabilitation is worth investigating. However, data on the relationship between sarcopenia and dysphagia in stroke are inconsistent [16,17,21], and the effect of sarcopenia on post-stroke recovery of swallowing function is inconclusive. Additionally, whether the adverse effects of sarcopenia on dysphagia can be improved by therapeutic approaches, such as nutritional support and rehabilitation training, remain unclear. This retrospective cohort study aimed to clarify whether sarcopenia impedes the recovery of deglutition function and whether the effects of sarcopenia are influenced by nutrition care and rehabilitation in stroke rehabilitation patients.

## 2. Materials and Methods

### 2.1. Data Collection

This retrospective multicenter cohort study was conducted in three hospitals (with 143–500 beds each) with convalescent rehabilitation wards and located in different prefectures in Japan: Hamamatsu City, Shizuoka; Daito City, Osaka; Nagasaki City, Nagasaki. All three hospitals included certified convalescent rehabilitation wards under public healthcare insurance policy in Japan [22,23]. Local investigators collected data for consecutive patients admitted to each hospital for post-stroke rehabilitation between 1 November 2018, and 31 October 2020. All patients were followed until discharge from hospital. All data were recorded using Excel and anonymized by the local investigators. After data collection, data for two sites (Hamamatsu and Daito) were sent to the principal investigator in Nagasaki. A complete dataset was created after data checking and cleaning the data.

Inclusion criteria were age ≥65 years and swallowing disorder determined as Food Intake Level Scale (FILS) of ≤7, indicating that a modified diet is required but no alternative nutrition [24]. Exclusion criteria were bulbar palsy, length of hospital stay of ≤7 days, inaccurate measurement with bioelectrical impedance analysis (BIA), such as pacemaker implantation, and inability to measure correct handgrip strength due to unconsciousness or quadriplegia as defined by ≤4 Brunnstrom stage.

The study was conducted according to the principles of the Declaration of Helsinki in 1964 and later amendments. The local ethics committee approved the study protocol (approval no. R3-1). Informed consent from individual participants was waived because of the retrospective study design, and all participants were given the opt-out option to withdraw from the study at any time.

### 2.2. Baseline Data

Data were collected from patients’ medical records, and the following patient characteristics were noted: age, sex, days from onset to admission, diagnosis (cerebral infarction, intracerebral hemorrhage, and subarachnoid hemorrhage), need of care before onset (confirmed by public long-term care insurance certification) [25], daily duration of physical, occupational, of speech-language-hearing rehabilitation (minutes/day), presence and severity of hemiplegia (Brunnstrom stage) [26], comorbidity (Charlson comorbidity index [CCI], updated) [27], activities of daily living (Functional Independence Measure [FIM]) [28], malnutrition risk (Malnutrition Universal Screening Tool [MUST]) [29], nutritional intake (average energy and protein intake/kg body weight/day), and swallowing abilities (determined using FILS) [24]. FILS is a 10-point observer-rating scale that was validated for the Functional Oral Intake Scale [24]. In this study, FILS level was assessed by a trained healthcare professional at each facility. Energy intake and duration of daily rehabilitation (minutes/day) were used as surrogate markers of nutritional support and rehabilitation therapy, respectively.

### 2.3. Sarcopenia Assessment

Sarcopenia was determined using the definition given by Asian Working Group on Sarcopenia (AWGS) 2019 [30]. After estimating appendicular muscle mass (kg) via BIA, the skeletal muscle mass index (SMI) was calculated as the appendicular muscle mass divided by height (m) squared. Cutoff values for low SMI were <7.0 kg/m^2^ for men and <5.7 kg/m^2^ for women. Handgrip strength was measured using a dynamometer, with low handgrip strength defined as <28 kg for men and <18 kg for women. Patients who had low SMI, as well as low handgrip strength, were considered to have sarcopenia. Assessment of physical function was not included in sarcopenia evaluation because some patients would have poor function, such as decreased walking speed, because of hemiplegia. Additionally, phase angle was obtained by BIA to assess cell membrane integrity.

### 2.4. Outcome Measures

The primary outcome was an FILS gain of ≥2. FILS levels can range between 1 (not performing swallowing training because of severe dysphagia or unconsciousness) and 10 (no problem with eating). Levels 1–3 indicate no oral intake, levels 4–6 indicate oral intake and alternative nutrition, and levels 7–10 indicate oral intake alone. The correlation coefficient (r) of FILS for the Functional Oral Intake Scale was reported as 0.96–0.99 [24]. An FILS gain of ≥2 was considered clinically important according to our previous study [31]. The secondary outcomes were FIM gain, calculated as FIM at discharge subtracted by FIM at admission, and FIM efficiency, calculated as FIM gain divided by length of hospital stay.

### 2.5. Sample Size Calculation

The required sample size was calculated using the Power and Sample Size Calculation software version 3.0 (William D. Dupont and Walton D. Plummer, Vanderbilt University School of Medicine, Nashville, TN, USA). As no data were available on the recovery of swallowing function in stroke rehabilitation patients with dysphagia and sarcopenia, we calculated the sample size on the basis of a previous study examining full oral intake recovery among stroke patients who had dysphagia with or without moderate malnutrion32). If the proportions of the regained ability of oral intake were 41.4% and 66.7% for patients with and without sarcopenia (25% difference) and the ratio of sarcopenic to non-sarcopenic patients was 3, then 159 participants (119 sarcopenic patients, 40 non-sarcopenic patients) would be required to obtain statistically significant results with an alpha error of 0.05 and power of 0.8. Considering the patients excluded in the analytic phase, we estimated that data of 200 patients would be required for this study.

### 2.6. Statistical Analyses

All statistical procedures were performed using Stata version 16 software (StataCorp, College Station, TX, USA). Normally distributed variables are expressed as the mean and standard deviation (SD), skewed distributed variables are presented as the median and interquartile range (IQR), and categorical variables are indicated as the number and percentage. Normality was confirmed via histograms. To compare the baseline characteristics and outcome measures between the patients with and without sarcopenia, unpaired *t*-test (for normal variables) and Wilcoxon rank-sum test (for skewed variables) were performed. Categorical variables were analyzed using chi-squared test and the Fisher exact test, followed by Bonferroni correction if there were >2 categories.

Logistic regression analysis was performed to adjust the confounding effects of the association between sarcopenia and FILS gain, FIM gain, and FIM efficiency. Age, sex, pre-stroke needs for care, primary disease (ischemic/hemorrhagic), CCI, FIM on admission, phase angle, and FILS on admission were used as potential confounders in the statistical model. Multicollinearity was confirmed if two variables had a correlation coefficient of ≥0.95 on Spearman’s rank correlation test. The FIM gain and efficiency were dichotomized by median values for these analyses. In addition, the interactions among rehabilitation duration and energy intake for sarcopenia were tested using interaction terms in logistic models. For this analysis, daily rehabilitation duration (min/day) and energy intake (kg body weight/day) were dichotomized by the median value. Statistical significance was set at *p* < 0.05.

## 3. Results

A total of 275 patients were eligible for data collection during the study period. Of these, 82 were excluded for the following reasons: 19 had bulbar palsy, 12 had missing BIA data, six had difficulties in the measurement of handgrip strength due to hemiplegia, four had rehabilitation ward stays ≤7 days, and 41 had no data on handgrip strength. Finally, 193 patients were included in the analysis.

Table 1 presents the patient characteristics. There were no missing values except for phase angle for one patient. Of 193 patients, 153 (79.3%) were identified as having sarcopenia and 40 (20.7%) were identified as without. Compared with non-sarcopenic patients, sarcopenic patients were significantly older and female, with a higher proportion of hemorrhagic stroke and certified need for care before stroke onset. In addition, sarcopenic patients had longer onset-to-admission duration, lower BMI, lower FIM, and poorer FILS score than their non-sarcopenic counterparts. Length of hospital stay, daily rehabilitation duration, and energy and protein intake were not significantly different between the groups.

At discharge, the FILS score of the non-sarcopenia group was significantly higher than that of the sarcopenia group (*p* < 0.001; Table 2). Of the 40 patients without sarcopenia, 75% had a FILS gain of ≥ 2, whereas 51.0% of the 153 patients with sarcopenia achieved the same degree of recovery for swallowing function (*p* = 0.006). Both FIM gain and efficiency were significantly higher in patients without sarcopenia than in those with. These associations persisted when these measures were dichotomized by median values (median FIM gain and efficiency, 22 and 0.27, respectively). Approximately 70% of non-sarcopenic patients and 49% of sarcopenic patients were discharged to their own home, although this finding did not reach statistical significance (*p* = 0.12).

Logistic regression analysis indicated that the sarcopenia group was associated with lower odds of meaningful FILS gain than the non-sarcopenia group (odds ratio [OR] = 0.35, *p* = 0.008; Table 3). There was no multicollinearity between the covariates included in the regression model. After adjustment for confounding effects, this association remained significant (adjusted OR = 0.34, 95% CI = 0.13–0.86). Conversely, there were little evidence against the null hypothesis stating that there was no association between sarcopenia and FIM gain and FIM efficiency (Table 4). Moreover, a test for interaction did not indicate statistically significant effect modification by rehabilitation duration (adjusted OR = 1.83, 95% CI = 0.34–10.2) and an energy intake (adjusted OR = 0.54, 95% CI = 0.10–2.98) for the association between sarcopenia and FILS gain (Tables S1 and S2).

## 4. Discussion

This multicenter retrospective cohort study revealed three important findings. First, many stroke patients had sarcopenia, and sarcopenia was independently associated with the recovery of deglutition function. Second, these associations were not affected by energy intake and rehabilitation duration. Third, sarcopenia did not affect the recovery of activities of daily living.

Our results indicated an association between sarcopenia and FILS gain, indicating the adverse effect of sarcopenia on recovery of deglutition function. This finding supports the results of previous studies that examined the negative relationship between sarcopenia or malnutrition and regaining deglutition function for stroke rehabilitation patients [16,17,32,33]. Skeletal muscle loss often occurs in stroke patients for many reasons, such as denervation, impaired efferent neurovegetative control, and dysphagia, also called stroke-related sarcopenia [18]. Although this concept supports the hypothesis that dysphagia after stroke can cause sarcopenia, our results may evoke the potential for reverse causality; sarcopenia may impede the recovery of dysphagia associated with stroke. Therefore, we hypothesize that dysphagia after stroke can be accompanied by sarcopenia, even though the recent diagnostic analysis for sarcopenic dysphagia excludes patients with post-stroke dysphagia [7]. In contrast, a cohort study presented opposite result, wherein sarcopenia was not associated with the FILS level at discharge in stroke rehabilitation patients [21]. This inconsistency may be partially explained by the outcome measures (FILS gain vs. FILS at discharge) and the target population (stroke patients with dysphagia vs. all stroke patients). Focusing on the recovery of swallowing function in post-stroke patients with dysphagia, we believe that sarcopenia can attenuate the effects of swallowing rehabilitation.

In this study, the association between sarcopenia and swallowing function was not affected by the amount of energy intake. This result is in contrast to previous case reports that indicate a positive effect of aggressive nutritional therapy for sarcopenic dysphagia in patients with lung cancer [34], pneumonia [35], and coronavirus disease 2019 [36]. Another cohort study indicated that provision of ≥30 kcal/kg ideal body weight was significantly associated with better FILS gain than that of <30 kcal/kg ideal body weight in patients with sarcopenic dysphagia [31]. A potential reason for this inconsistency is that not all patients with sarcopenia exhibit sarcopenic dysphagia; therefore, nutritional therapy may not aid in the recovery from dysphagia among stroke patients without sarcopenic dysphagia. Furthermore, we found that the daily rehabilitation duration (min/day) did not indicate an interaction between sarcopenia and recovery of swallowing function. Swallowing rehabilitation is apparently an effective treatment for post-stroke dysphagia [5]. However, the type and extent of swallowing rehabilitation that may be beneficial to patients with sarcopenic dysphagia remain unknown. Further investigation is required to clarify the usefulness of swallowing rehabilitation and its duration for stroke patients with sarcopenic dysphagia.

Our study revealed that sarcopenia did not affect FIM gain and efficiency. To date, studies have reported a negative effect of sarcopenia on the outcomes for activities of daily living in post-stroke patients [19,21,37]. A possible explanation for this discrepancy is the extensively high prevalence of sarcopenia in our study (79.3%) compared with that in previous studies (42–52%) [19,21,37], which may have diluted the adverse effect of sarcopenia. An alternative explanation may be the variety of outcome measures; our study used dichotomized FIM gain and efficiency, whereas other studies used the FIM motor score at discharge [19,21] and poor functional recovery defined by the modified Rankin scale [37]. Body composition assessment might also be crucial to predicting the physical and mental quality of life in older adults [38,39].

This study had several limitations. Firstly, we could not distinguish sarcopenia originating before stroke onset from that after stroke. According to its etiology, sarcopenia before and after stroke may be induced by different causes; therefore, the possible effects on swallowing function may also differ. We speculate that stroke patients who have sarcopenia before stroke onset are more likely to have sarcopenic dysphagia. More research is needed to specify the onset of sarcopenia in stroke patients. Secondly, we did not define sarcopenic dysphagia using a diagnostic algorithm [15]. Thus, the prevalence and effect of sarcopenic dysphagia in stroke patients cannot be determined in our study. Thirdly, the measure of swallowing function, FILS, did not directly indicate swallowing function. It is desirable to use more precise methods, such as videofluoroscopic examination, for investigating the effect of sarcopenia on swallowing function. Fourthly, we could not collect data on stroke severity, which is strongly correlated with dysphagia, due to limited information from the acute care hospitals. Instead, we adjusted the confounding effect of the FIM that is associated with stroke severity [40]. Lastly, we could not gather information on pre-stroke swallowing function, which would be a confounder for the prognosis of dysphagia. To overcome this limitation, we adjusted the confounding effect of pre-stroke need for care that may relate to swallowing function.

## 5. Conclusions

We found a negative association between sarcopenia and recovery of deglutition function in patients during post-stroke rehabilitation, and this association was not influenced by energy intake and rehabilitation duration. These results highlight the fact that some stroke patients with dysphagia may have sarcopenic dysphagia. Although the cause–effect relationship between sarcopenia and dysphagia was not established in our study, it is worth investigating whether stroke patients can be diagnosed with sarcopenic dysphagia using a diagnostic algorithm and, if so, what interventions are effective for these patients.

## Figures and Tables

**Table 1:** Baseline characteristics of 193 stroke patients with dysphagia in convalescent rehabilitation wards.

	Non-Sarcopenia (*n* = 40)	Sarcopenia (*n* = 153)	*p*-Value
Female, *n* (%)	14 (35.0%)	88 (57.5%)	0.011
Age, years	75 (72–78.5)	82 (76–86)	<0.001
Onset-to-admission duration, days	23.5 (16–30)	27 (21–39)	0.018
Length of rehabilitation ward stay	89 (64–111)	93 (64–126)	0.26
Diagnosis			
Cerebral infarction	32 (80.0%)	94 (61.4%)	0.045
Intracerebral hemorrhage	8 (20.0%)	46 (30.1%)	
Subarachnoid hemorrhage	0 (0.0%)	13 (8.5%)	
Pre-stroke need for care, n (%)	4 (10.0%)	41 (26.8%)	0.025
Daily rehabilitation dose (min/day)	163 (155–171)	162 (150–178)	0.97
Hemiplegia			
None	9 (22.5%)	20 (13.1%)	0.37
Right	13 (32.5%)	62 (40.5%)	
Left	17 (42.5%)	62 (40.5%)	
Bilateral	1 (2.5%)	9 (5.9%)	
CCI updated, points	1 (1–2)	1 (1–2)	0.76
Height, cm, mean (SD)	159.2 (9.1)	154.3 (8.5)	0.001
Weight, kg, mean (SD)	58.6 (10.0)	48.1 (8.8)	<0.001
BMI, kg/m^2^, mean (SD)	23.1 (3.2)	20.2 (3.0)	<0.001
FIM motor score, points	42.5 (36.5–53)	25 (15–37)	<0.001
FIM cognitive score, points	23 (17–28.5)	16 (12–22)	0.001
FIM total score, points	66.5 (55.5–77.5)	42 (29–57)	<0.001
FILS, level	7 (7–7)	7 (3–7)	0.022
SMI, kg/m^2^	6.8 (6.0–7.2)	5.0 (4.3–5.8)	<0.001
Phase angle, °, mean (SD) *	4.3 (0.9)	3.5 (0.8)	<0.001
Handgrip strength, kg	26.3 (18.5–30.2)	11.6 (8.2–16.4)	<0.001
MUST score, points	1.5 (0–3)	2 (1–4)	0.001
Energy intake, kcal/day, mean (SD)	25.4 (6.1)	26.3 (8.4)	0.53
Protein intake, g/day, mean (SD)	1.0 (0.2)	1.1 (0.4)	0.18

Values expressed as the median (interquartile range) unless specified otherwise. BMI, body mass index; CCI, Charlson comorbidity index; FIM, Functional Independence Measure; MUST, Malnutrition Universal Screening Tool; SD, standard deviation; SMI, skeletal muscle mass index. * One datum was missing.

**Table 2:** FILS and discharge outcomes for 193 stroke rehabilitation patients with dysphagia.

	Non-Sarcopenia (*n* = 40)	Sarcopenia (*n* = 153)	*p*-Value
FILS, points	9 (8–9)	8 (7–9)	<0.001
FILS gain ≥ 2, *n* (%)	30 (75.0%)	78 (51.0%)	0.006
FIM motor score, points	76.5 (63–84)	50 (31–66)	<0.001
FIM cognitive score, points	26.5 (22.5–32)	20 (15–27)	<0.001
FIM total score, points	105 (83.5–113)	70 (48–89)	<0.001
FIM gain			
Points	33.5 (19.5–42)	22 (12–35)	0.021
>22 points, *n* (%) *	29 (72.5%)	74 (48.4%)	0.006
FIM efficiency			
Points/day	0.34 (0.22–0.48)	0.26 (0.15–0.37)	0.013
>0.27 points/day, *n* (%) *	26 (65.0%)	71 (46.4%)	0.036
BMI, kg/m^2^, mean (SD)	22.6 (2.9)	20.3 (2.7)	<0.001
Discharge destination, n (%)			0.12
Home	28 (70.0%)	75 (49.0%)	
Long-term care facilities	11 (27.5%)	56 (36.6%)	
Long-term care hospitals	0 (0%)	7 (4.6%)	
Acute care hospitals	1 (2.5%)	14 (9.2%)	
Death	0 (0%)	1 (0.7%)	

Values expressed as the median (interquartile range) unless specified otherwise. BMI, body mass index; FILS, Food Intake LEVEL Scale; FIM, Functional Independence Measure; SD, standard deviation. * Cutoff values were defined by median in the entire sample.

**Table 3:** Logistic regression analysis for FILS gain (≥2 points).

	OR		95% CI for AOR		*p*-Value
	Crude	Adjusted	Lower	Upper	
Age	0.94	0.97	0.92	1.03	0.35
Female sex	0.65	0.89	0.41	1.93	0.77
Pre-stroke need for care	0.55	0.70	0.31	1.61	0.41
CCI, updated	0.72	0.67	0.50	0.91	0.010
FIM total score	1.00	1.01	0.99	1.02	0.47
FILS score	0.73	0.68	0.56	0.83	<0.001
Hemorrhage stroke	1.53	1.13	0.53	2.40	0.75
Phase angle	1.59	1.24	0.74	2.07	0.42
Sarcopenia	0.35	0.34	0.13	0.86	0.023

AOR, adjusted odds ratio; CCI, Charlson comorbidity index; FILS, Food Intake Level Scale; FIM, Functional Independence Measure; OR, odds ratio. A total of 192 patients were included, excluding one patient with a missing phase angle value.

**Table 4:** Logistic regression analysis for FIM gain and FIM efficiency **.

	OR		95% CI for AOR		*p*-Value
	Crude	Adjusted	Lower	Upper	
FIM gain (>22 points) *					
Age	0.94	0.97	0.92	1.01	0.21
Female sex	0.66	0.79	0.38	1.64	0.55
Pre-stroke need for care	0.34	0.53	0.24	1.16	0.11
CCI, updated	0.77	0.74	0.56	0.97	0.031
FIM total score	1.00	0.99	0.98	1.01	0.25
FILS score	1.08	1.14	0.96	1.34	0.14
Hemorrhage stroke	1.02	1.01	0.49	2.07	0.97
Phase angle	1.68	1.30	0.80	2.10	0.29
Sarcopenia	0.36	0.63	0.27	1.46	0.28
FIM efficiency (>0.27 points/day) *					
Age	0.99	1.00	0.95	1.05	1.00
Female sex	0.79	0.97	0.48	1.97	0.93
Pre-stroke need for care	0.74	0.87	0.41	1.85	0.72
CCI, updated	0.93	0.92	0.71	1.19	0.51
FIM total score	1.02	1.02	1.00	1.03	0.045
FILS score	1.15	1.08	0.92	1.27	0.37
Hemorrhage stroke	0.65	0.74	0.37	1.50	0.41
Phase angle	1.30	1.12	0.71	1.75	0.64
Sarcopenia	0.47	0.78	0.34	1.82	0.57

AOR, adjusted odds ratio; CCI, Charlson comorbidity index; FILS, Food Intake Level Scale; FIM, Functional Independence Measure; OR, odds ratio. * Defined by median value in entire sample. ** Data on one patient with missing phase angle value were excluded (*n* = 192).

## Data Availability

Not applicable.

## Supplementary Materials

The following supporting information can be downloaded at https://www.mdpi.com/article/10.3390/nu14194115/s1: Table S1. Logistic regression analysis for FILS gain (≥2 points) including interaction between daily rehabilitation dose and sarcopenia; Table S2. Logistic regression analysis for FILS gain (≥2 points) including interaction between energy intake and sarcopenia.
