# Supplementary material for: Association of Existence of Sarcopenia and Poor Recovery of Swallowing Function in Post-Stroke Patients with Severe Deglutition Disorder: A Multicenter Cohort Study
Source: Nutrients. 2022 Oct 3;14(19):4115. doi: 10.3390/nu14194115 (PMC9571320; doi:10.3390/nu14194115)
Supplement: Supplementary file 1 [file nutrients-14-04115-s001.zip › nutrients-1859958-supplementary.pdf]

Table S1. Logistic regression analysis for FILS gain (≥2 points) including interaction between daily rehabilitation dose and sarcopenia

	AOR	95% CI		P value
		Lower	Upper	
Age	0.96	0.91	1.01	0.13
Female sex	1.06	0.49	2.30	0.89
CCI, updated	0.70	0.52	0.95	0.023
FIM total score	1.01	0.99	1.02	0.35
FILS score	0.66	0.54	0.81	<0.001
Phase angle	1.22	0.73	2.06	0.45
Daily rehabilitation dose (≥162 min/day)*	1.11	0.23	5.29	0.90
Sarcopenia	0.25	0.07	0.91	0.035
Rehabilitation dose × sarcopenia	1.86	0.34	10.2	0.47

AOR, adjusted odds ratio; CCI, Charlson comorbidity index; FILS, Food Intake LEVEL Scale; FIM, Functional Independence Measure; OR, odds ratio. *Defined by median value in entire sample

Table S2. Logistic regression analysis for FILS gain (≥2 points) including interaction between energy intake and sarcopenia

	AOR	95% CI		P value
		Lower	Upper	
Age	0.96	0.92	1.01	0.16
Female sex	0.92	0.43	1.95	0.82
CCI, updated	0.66	0.49	0.89	0.006
FIM total score	1.01	0.99	1.02	0.47
FILS score	0.68	0.56	0.82	<0.001
Phase angle	1.20	0.71	2.02	0.50
Energy intake (≥26 kcal/day)*	1.42	0.31	6.59	0.65
Sarcopenia	0.45	0.13	1.58	0.21
Energy intake × sarcopenia	0.54	0.10	2.98	0.48

AOR, adjusted odds ratio; CCI, Charlson comorbidity index; FILS, Food Intake LEVEL Scale; FIM, Functional Independence Measure; OR, odds ratio. *Defined by median value in entire sample
